# Mechanical stretch regulates macropinocytosis in *Hydra vulgaris*

**DOI:** 10.1091/mbc.E22-02-0065

**Published:** 2024-02-02

**Authors:** Taylor D. Skokan, Bert Hobmayer, Kara L. McKinley, Ronald D. Vale

**Affiliations:** a Howard Hughes Medical Institute and Department of Cellular and Molecular Pharmacology, University of California, San Francisco, San Francisco, CA 94158; b Department of Zoology and Centre for Molecular Biosciences Innsbruck (CMBI), University of Innsbruck, Technikerstr. 25, A-6020 Innsbruck, Austria; c Howard Hughes Medical Institute and Department of Stem Cell and Regenerative Biology, Harvard University, Cambridge, MA 02138; d Howard Hughes Medical Institute Janelia Research Campus, Ashburn, VA, 20147; King’s College London

## Abstract

Cells rely on a diverse array of engulfment processes to sense, exploit, and adapt to their environments. Among these, macropinocytosis enables indiscriminate and rapid uptake of large volumes of fluid and membrane, rendering it a highly versatile engulfment strategy. Much of the molecular machinery required for macropinocytosis has been well established, yet how this process is regulated in the context of organs and organisms remains poorly understood. Here, we report the discovery of extensive macropinocytosis in the outer epithelium of the cnidarian *Hydra vulgaris*. Exploiting *Hydra*’s relatively simple body plan, we developed approaches to visualize macropinocytosis over extended periods of time, revealing constitutive engulfment across the entire body axis. We show that the direct application of planar stretch leads to calcium influx and the inhibition of macropinocytosis. Finally, we establish a role for stretch-activated channels in inhibiting this process. Together, our approaches provide a platform for the mechanistic dissection of constitutive macropinocytosis in physiological contexts and highlight a potential role for macropinocytosis in responding to cell surface tension.

## Abstract (executive summary)

Macropinocytosis is a versatile endocytic strategy involved in nutrient acquisition, immune surveillance, and membrane remodeling. Despite extensive molecular characterization, much remains unknown about the regulation of macropinocytosis in tissues.

The authors report a previously undescribed form of constitutive macropinocytosis in the epithelium of *Hydra* and reveal a role for tissue stretch in regulating this process.

Our findings suggest that constitutive macropinocytosis may be more pervasive than previously appreciated and highlight a potential role for this biological phenomenon in membrane tensioning and tissue remodeling.

## INTRODUCTION

Engulfment processes are essential for cells to sense and interact with their extracellular environments. In contrast to receptor-mediated endocytosis and phagocytosis, which rely on interactions with specific ligands, macropinocytosis provides a mechanism to indiscriminately engulf large volumes of extracellular fluid and plasma membrane in the absence of a defined target (Kerr and Teasdale, 2009). This unbiased strategy renders macropinocytosis a highly versatile process, illustrated by the diverse functions it serves (reviewed in Bloomfield and Kay, 2016), including bulk nutrient acquisition (Commisso *et al.*, 2013; Bloomfield *et al.*, 2015), immune surveillance (Sallusto *et al.*, 1995; Sarkar *et al.*, 2005), receptor sequestration (Orth *et al.*, 2006), and membrane retrieval (Holt *et al.*, 2003; Clayton *et al.*, 2008). Regardless of the specific cellular functions served by macropinocytosis, the process involves local remodeling of the actin cytoskeleton to form membrane protrusions, or ruffles, that subsequently fuse to encapsulate extracellular contents. During this process, formation of membrane ruffles depends on the temporally and spatially restricted interactions of phosphoinositides, small GTPases, and actin regulators (reviewed in Buckley and King, 2017).

Despite extensive molecular characterization, the factors that initiate macropinocytosis in diverse cell types and physiological contexts are incompletely understood. In some cell types, most notably antigen presenting cells, macropinocytosis occurs constitutively, whereas many other cell types require exogenous growth factor stimulation or pathogen exposure to stimulate macropinocytosis (West *et al.*, 1989; Rosales-Reyes *et al.*, 2012). Recent work has identified factors that can enhance the rate of growth factor-mediated macropinocytosis, including excessive cortical localization of the cytoskeleton-membrane linker ezrin (Chiasson-MacKenzie *et al.*, 2018). Moreover, recent work in mammalian myotubes revealed that transient reductions in membrane tension following osmotic stress and mechanical stretch can promote growth factor-stimulated macropinocytosis (Lin and Liu, 2019; Loh *et al.*, 2019).

Here, we report the serendipitous discovery of tissue-wide macropinocytosis in the freshwater polyp *Hydra vulgaris*, a representative of the ancestral animal phylum Cnidaria. In contrast to canonical, mammalian models of epithelial macropinocytosis, which require growth factor stimulation (reviewed in Lin *et al.*, 2020), this process unfolds constitutively in *Hydra*’s superficial ectodermal epithelium, accounting for considerable membrane remodeling and fluid uptake. Combining live microscopy with pharmacological perturbations, we establish a role for stretch-activated channels and Ca^2+^ signaling in regulating this process. Finally, by inflating regenerating *Hydra* tissues, we demonstrate a direct role for cellular stretch in regulating macropinocytosis, with high-tension states inhibiting fluid uptake. Together, our findings reveal a role for tissue mechanics in regulating constitutive macropinocytosis and highlight the potential for *Hydra* as a physiological model of macropinocytosis.

## RESULTS

### *Hydra* ectoderm exhibits ubiquitous macropinocytosis

**FIGURE 1:**
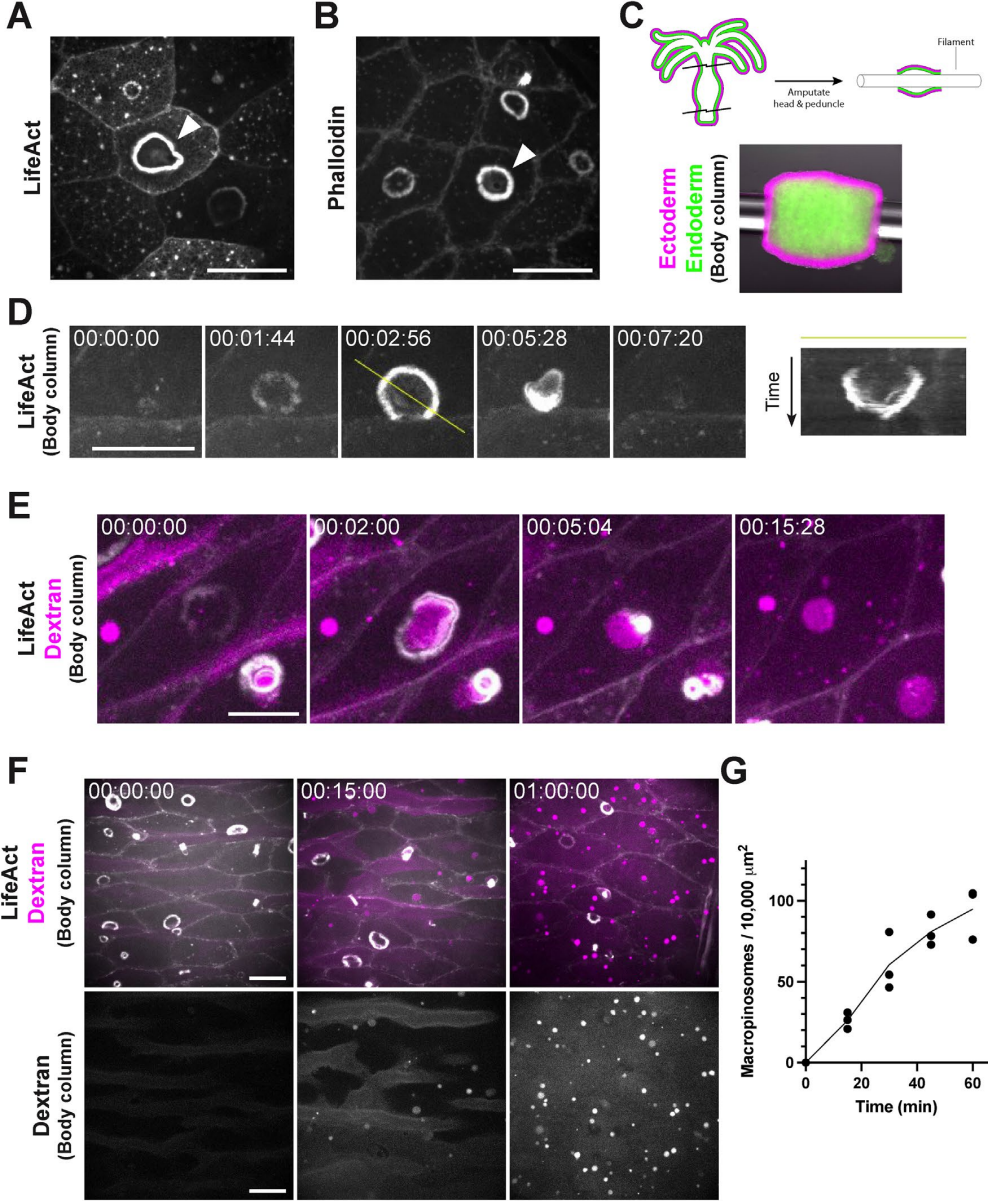
*Hydra* ectoderm exhibits ubiquitous macropinocytosis. A) Representative image of macropinocytic cups (arrowhead) in the ectoderm of a live, intact, LifeAct-GFP-expressing *Hydra*. B) Representative image of macropinocytic cups (arrowhead) in the ectoderm of a fixed, intact *Hydra* stained with phalloidin. C) Schematic (top) and representative image (bottom) of the threaded body column preparation used for prolonged live imaging. Ectodermal and endodermal cells express DsRed2 (magenta) and GFP (green), respectively. D) Representative time-course of macropinocytic cup formation, closure, and dissipation, visualized by LifeAct-GFP (left). Image registration was performed to compensate for translational movement in the body column. Yellow line denotes the axis of the corresponding kymograph showing the complete lifecycle (right). E) Representative time-course of fluorescently labeled dextran (magenta) engulfment by macropinocytic cups, visualized by LifeAct-GFP (white). Image registration was performed to compensate for translational movement in the body column. F) Representative time-course of dextran-filled macropinosome (top, magenta; bottom, white) accumulation in ectodermal tissues expressing LifeAct-GFP (white). G) Quantification of dextran-filled macropinosome accumulation shown in (F) (mean ± sd; *n* = 3 independent sample preparations). (A–F) All frames depict maximum intensity projections of 10–35 μm z-stacks. Time stamps, hh:mm:ss. Scale bars, 20 μm. (D–F) All time-courses depict threaded body columns.

Previous investigations of tissue patterning in *Hydra* have shown that the actin reporter LifeAct-GFP localizes to actin-enriched basal myonemes and apical cell junctions of the outer (ectodermal) epithelium (Aufschnaiter *et al.*, 2017; Skokan *et al.*, 2020). In our studies, we also observed that LifeAct-GFP labeled dynamic, ring-shaped structures that localized to the apical membrane of epithelial cells (Figure 1A). Phalloidin staining in fixed, intact animals confirmed that these structures were enriched for actin (Figure 1B). These actin-rich rings were found at a low frequency along the entire body (Supplemental Figure S1A) and bore a striking resemblance to structures identified in classic scanning electron microscopy of the *Hydra* ectoderm (Beams *et al.*, 1973). Because *Hydra* displays considerable movement in three-dimensions, we developed a preparation that would allow us to better monitor the dynamics of the actin rings over extended periods. This preparation involved amputating the head and foot and threading a nylon filament through the body column to roughly constrain tissue movement to a single plane (Figure 1C). This approach allowed us to capture the entire lifecycle of actin rings, which form as rings, initially expand (maximum diameter = 10.49 ± 2.6 μm; *n* = 35 from three independent preparations), and then constrict to a focus and disappear (lifespan: 109 ± 32 s; *n* = 35 from three independent preparations; Figure 1D; Supplemental Video S1A).

**Figure:** Video S1 A) Representative time-course of macropinocytic cup formation, closure, and dissipation, visualized by LifeAct-GFP. Image registration was performed to compensate for translational movement in the body column. Corresponds to Figure 1D. B) Representative time-course of fluorescently labeled dextran (magenta) engulfment by macropinocytic cups, visualized by LifeAct-GFP (white). Image registration was performed to compensate for translational movement in the body column. Corresponds to Figure 1E. (A, B) All videos depict maximum intensity projections of 10-50 μm z-stacks. Time stamps, hh:mm:ss.

Given their resemblance to circular dorsal ruffles and macropinocytic cups in mammalian cells (Mellström *et al.*, 1983; reviewed in Buccione *et al.*, 2004), we considered whether the ring structures may play a role in fluid uptake. To test this, we incubated threaded body columns in media containing fluorescently labeled dextran. As rings formed, dextran accumulated in the resulting invaginations and was engulfed upon ring constriction (Figure 1E; Supplemental Video S1B). The resulting dextran-filled vacuoles persisted within ectodermal cells and accumulated in the tissue over time (Figure 1, F and G). Thus, the actin rings denote macropinocytic cups associated with fluid uptake.

### Stretch-activated channel activity regulates macropinocytosis

**FIGURE 2:**
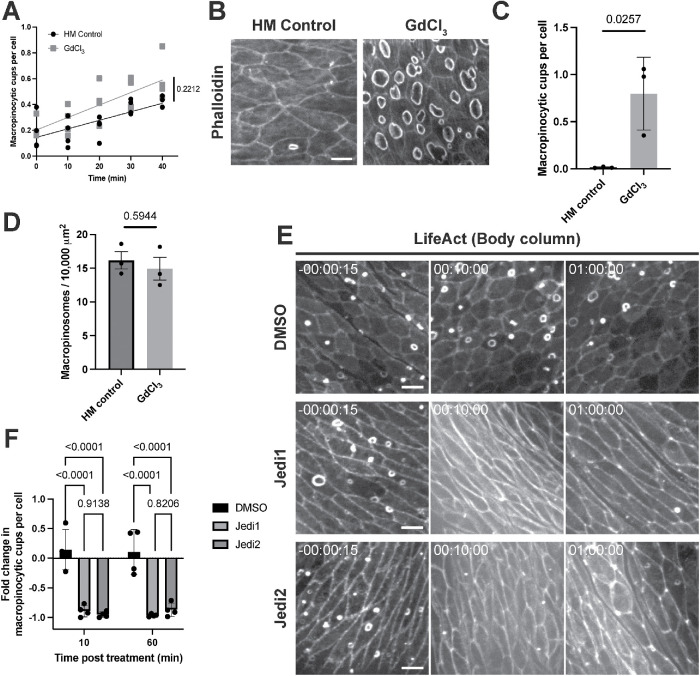
Stretch-activated channel activity regulates macropinocytosis. A) Quantification of macropinocytic cup abundance over time in *Hydra* medium (HM) control (left) and GdCl_3_-treated threaded body columns with fitted regression lines. The *t* = 0 min corresponds to the earliest acquirable time point after body column resection and threading (mean ± sd; *n* = 4 independent sample preparations per condition; linear regression slopes compared by ANCOVA). B) Representative images of macropinocytic cups stained with phalloidin in *Hydra* medium (HM) control (left) and GdCl_3_-treated (right; 50 μM for 15 min) intact *Hydra*. Scale bar, 20 μm. C) Quantification of macropinocytic cup frequency in response to treatments shown in (B). Bars indicate the mean ± sd of means from three independent sample preparations (represented by points); *n* ≥ 5 animals per preparation per condition (nested *t* test). D) Quantification of dextran-filled macropinosomes in control and GdCl_3_-treated intact *Hydra.* Bars indicate the mean ± sd of means for three independent sample preparations (represented by points); *n* ≥ 10 animals per preparation per condition (nested *t* test). E) Representative time-courses of threaded body columns expressing LifeAct-GFP in the ectoderm before (left) and after (center, right) treatment with DMSO, Jedi1 (200 μM), or Jedi2 (200 μM). Time stamps indicate time relative to drug addiction; hh:mm:ss. Scale bars, 20 μm. F) Quantification of change in macropinocytic cup frequency (fold change relative to pretreatment values) 10 or 60 min after specified treatments shown in (E) (Bars: mean ± sd; *n* = 4 independent sample preparations per condition; two-way ANOVA with Tukey’s multiple comparisons test). (B and E) All frames depict maximum intensity projections of 15–35 μm z-stacks. The *p*-values are displayed with corresponding comparisons.

Intriguingly, we observed more macropinocytic cups in isolated *Hydra* body columns (0.186 ± 0.140 cups per cell; *n* = 4 independent preparations) when compared with fixed, intact animals (0.015 ± 0.011 cups per cell; *n* = 15 from three independent preparations; Supplemental Figure S1B). Moreover, the frequency of macropinocytic cups increased over time in body columns following resection (Figure 2A; Supplemental Video S2A). As the incisions required for head and foot amputation are likely to affect tension in the epithelium, we considered whether tissue mechanics may play a role in regulating macropinocytosis.

**Figure:** Video S2 A) Representative time-course of macropinocytic cup frequency in a control isolated body column treated with *Hydra* medium (left) or GdCl_3_ (50 μM; right), visualized by LifeAct-GFP. Corresponds to Figure 2A. B) Representative time-courses of macropinocytic cup frequency in isolated body columns treated with DMSO (top-left), Jedi1 (200 μM; top-right), Jedi2 (200 μM; bottom-left), visualized by LifeAct-GFP. Corresponds to Figures 2E, 2F. (A-D) All videos depict maximum intensity projections of 35-100 μm z-stacks. Time stamps indicate time relative to drug addition; hh:mm:ss.

Stretch-activated channels contribute to tension sensing in a variety of biological contexts (Stewart and Davis, 2019), and analysis of recent *Hydra* single-cell RNA sequencing data (Siebert *et al.*, 2019) revealed that the *Hydra* ectoderm expresses several putative TRP (e.g., t27236aep, t29007aep) and Piezo (t21136aep) channel proteins that may play a role in mechanosensation. To determine whether stretch-activated channels modulate macropinocytic cup formation, we treated intact *Hydra* with gadolinium chloride (GdCl_3_), a broad-spectrum inhibitor of stretch-activated channels (Yang and Sachs, 1989). Immunofluorescence and live imaging in intact animals revealed a striking increase in macropinocytic cups following treatment with GdCl_3_ (50 μM; Figure 2, B and C; Supplemental Figure S2). Dextran uptake assays showed no increase in dextran-filled macropinosomes in the presence of GdCl_3_, suggesting that the increase in macropinocytic cups we observed upon GdCl_3_ treatment does not translate to an increase in productive engulfment events (Figure 2D). We observed no significant increase in the frequency of macropinocytic cups in GdCl_3_-treated threaded body columns over their already elevated levels of macropinocytosis (Figure 2A; Supplmental Video S2A), supporting the notion that the higher levels of macropinocytosis observed in this preparation may indeed result from mechanical unloading. To further probe the role of stretch-activated channels, we treated *Hydra* body columns with Jedi1 or Jedi2, activators of the stretch-activated channel Piezo1 (Wang *et al.*, 2018). Treatment with either Jedi1 or Jedi2 resulted in a near complete, albeit transient, depletion of macropinocytic cups in isolated body columns, coinciding with contraction of the tissue (Figure 2, E and F; Supplemental Video S2B). Together, these findings implicate stretch-activated channels in the regulation of macropinocytosis in *Hydra*.

### Mechanical stretch inhibits macropinocytosis

**FIGURE 3:**
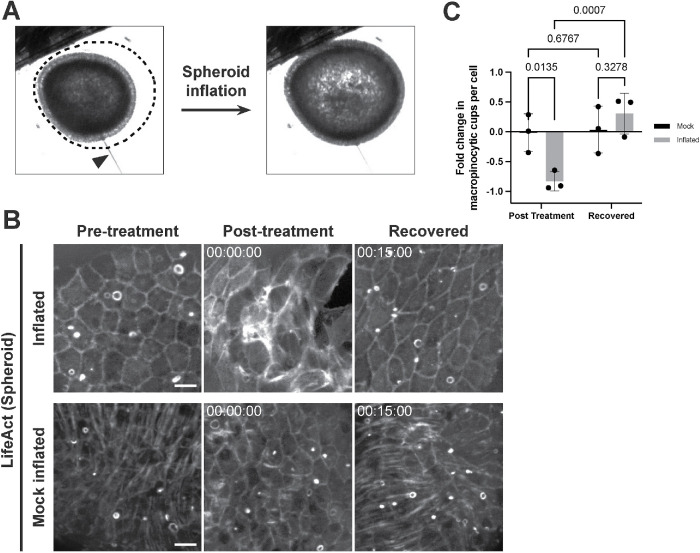
Application of tissue stretch inhibits macropinocytosis. A) Representative images of the same *Hydra* spheroid before (left) and after inflation (right). Dotted line depicts the profile of the spheroid at peak inflation. Arrowhead: microinjection needle. B) Representative time-course of the same spheroids expressing ectodermal LifeAct-GFP before (Pretreatment), immediately after inflation or mock inflation and needle removal (Post-treatment), and during recovery (Recovered). Time stamps indicate time relative to needle removal following inflation/mock inflation; hh:mm:ss. All frames depict maximum intensity projections of 15–35 μm z-stacks. Preinflation and Inflated/Recovered images have been scaled independently to compensate for an increase in signal following inflation. Scale bar, 20 μm. C) Quantification of change in macropinocytic cup frequency (fold change relative to pretreatment values) during treatments shown in (B) (mean ± sd; *n* = 3 independent sample preparations per condition; two-way ANOVA with uncorrected Fisher’s LSD multiple comparisons test). The *p* values are displayed with corresponding comparisons.

We next sought to directly test the effects of tissue stretch on macropinocytosis. Body column fragments that are allowed to heal (i.e., without threading onto filaments) form hollow tissue spheres, which undergo cycles of swelling and rupturing as they progress toward whole body regeneration (Shimizu *et al.*, 1993; Sato-Maeda and Tashiro, 1999). We sought to capitalize on the architectural simplicity and deformability of these regenerating “spheroids” as a system for applying stretch. To this end, we microinjected medium into the lumen of *Hydra* spheroids and were able to inflate them to ∼1.5 times their original volume without rupture (approximated volume fold change: 1.47 ± 0.19; Figure 3A; Supplemental Figure S3; Supplemental Video S3A). In an inflated state, ectodermal cells of LifeAct-GFP-expressing spheroids exhibited enlarged apical surface areas, suggestive of a planar ectodermal stretch (Figure 3B). Using this approach, we characterized the abundance of macropinocytic cups in inflated and uninflated LifeAct-GFP-expressing spheroids. Macropinocytic cups were depleted in inflated aggregates when compared with their preinflation states. As aggregates gradually deflated following needle removal, we observed a recovery of macropinocytic cups (Figure 3, B and C; Supplemental Video S3B). Importantly, mock inflation, in which a microinjection needle was inserted into a spheroid without inflation, did not affect macropinocytosis (Figure 3, B and C; Supplemental Video S3B).

**Figure:** Video S3 A) Representative time-course of spheroid inflation. Corresponds to Figure 3A. B) Representative time-course of macropinocytic cup frequency in spheroids imaged before (pre-puncture; top) and after mock inflation (left) or inflation (right), visualized by LifeAct-GFP. Note “pre-puncture” frames depict still images (single time point). Corresponds to Figures 3B, 3C. Videos in (B) depict maximum intensity projections of 35-100 μm z-stacks. Time stamp indicates time relative to needle removal following treatment; hh:mm:ss.

### Calcium influx is sufficient, but not necessary, to inhibit macropinocytosis

**FIGURE 4:**
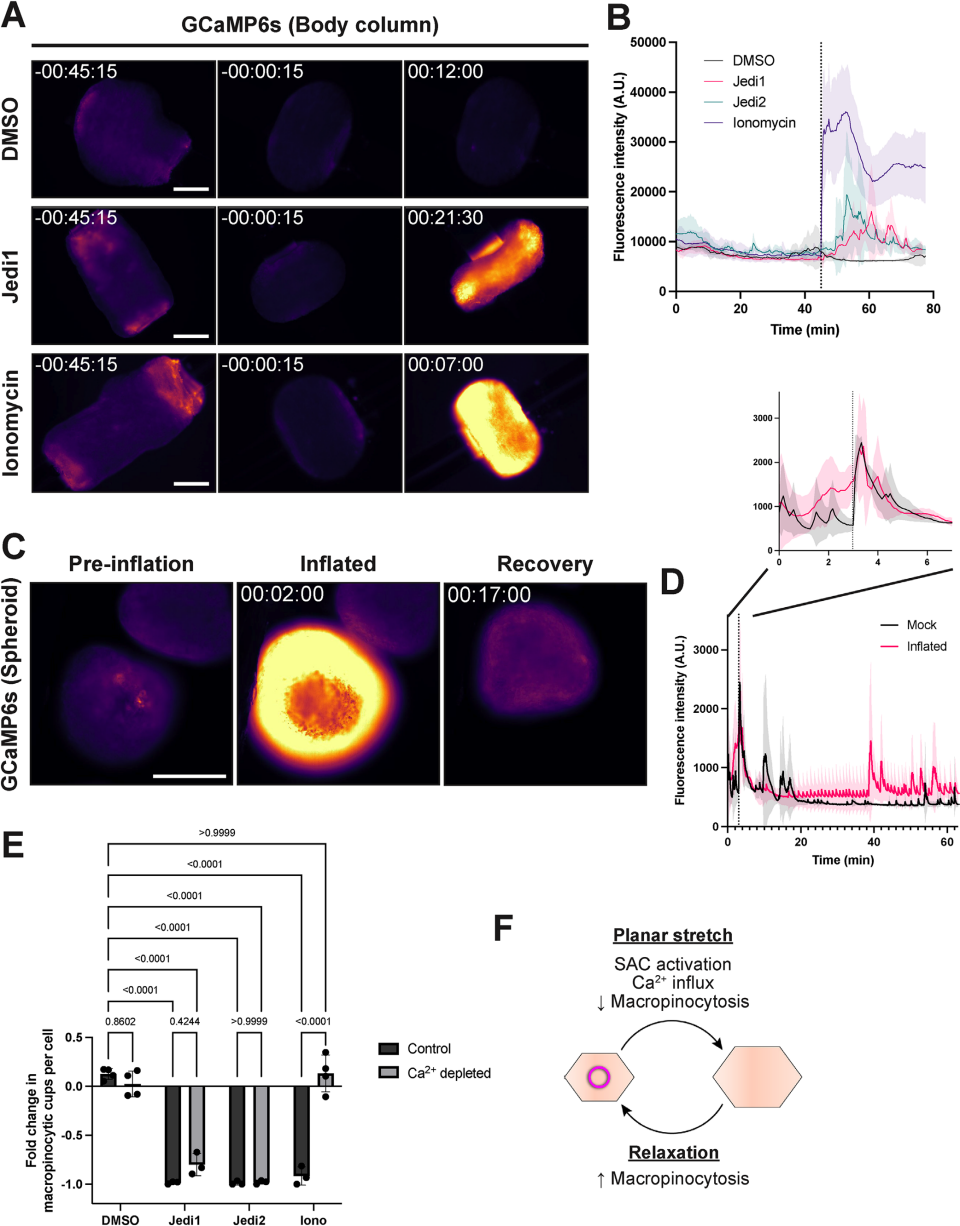
Calcium transients associated with inhibition of macropinocytosis. A) Representative time-courses of threaded body columns expressing GCaMP6s in the ectoderm, before (left, center) and after (right) DMSO, Jedi1 (200 μM), or Ionomycin (calcium salt, 10 μM) treatment. Time stamps indicate time relative to drug addiction; hh:mm:ss. mpl-inferno LUT (FIJI) was applied to aid in visualizing graded GCaMP6s signal. Scale bar, 500 μm. B) Traces depicting mean fluorescence intensity (arbitrary units) over time in threaded body columns before and after specified treatment (line and shading: mean ± sd; *n* = 4 independent sample preparations per condition). Dotted line indicates drug or vehicle addition. C) Representative time-course of the same spheroid expressing ectodermal GCaMP6s before (Preinflation), immediately after inflation (Inflated), and during recovery (Recovery). mpl-inferno LUT (FIJI) was applied to aid in visualizing graded GCaMP6s signal. Time stamp indicates time relative to initiation of inflation; hh:mm:ss. Scale bar, 200 μm. D) Traces depicting mean GCaMP6s fluorescence intensity over time during inflation experiments (line and shading: mean ± sd; *n* = 3 independent sample preparations per condition). Dotted line indicates needle removal. E) Quantification of change in macropinocytic cup frequency (fold change relative to pretreatment values) in isolated body columns in response to DMSO, Jedi1 (200 μM), Jedi2 (200 μM), or Ionomycin (free acid, 10 μM) treatment in control or calcium-depleted conditions (Bars: mean ± sd; *n* ≥ 3 independent sample preparations per condition; two-way ANOVA with Tukey’s multiple comparisons test). F) Model of mechanically regulated macropinocytosis in *Hydra*. Planar stretch results in stretch-activated channel (SAC) activation, calcium influx, and inhibition of macropinocytosis. Following tissue relaxation, SAC inactivation restores macropinocytosis. The *p* values are displayed with corresponding comparisons.

Given these findings, we sought to probe the downstream effects of mechanical stretch and stretch-activated channel activation in regulating macropinocytosis. Upon activation, stretch-activated channels signal mechanical stimuli through the transport of ions, including Ca^2+^ in the case of Piezo channels (Coste *et al.*, 2010). To directly monitor calcium flux in the *Hydra* ectoderm, we prepared threaded body columns from transgenic *Hydra* expressing the fluorescent calcium reporter GCaMP6s in ectodermal tissues (Szymanski and Yuste, 2019). We observed a gradual decrease in fluorescence intensity as body columns conformed to their filaments (Figure 4, A and B; Supplemental Video S4A), coinciding with the period of increasing macropinocytosis reported above. Treatment of body columns with Jedi1 or Jedi2 induced a transient spike in GCaMP6s fluorescence, accompanied by strong contractions (Figure 4, A and B; Supplemental Video S4A). Applying our inflation approach to GCaMP6s-expressing spheroids, we observed a similar increase in GCaMP6s fluorescence during spheroid inflation when compared with mock inflated spheroids, for which GCaMP6s intensity spiked only momentarily during needle insertion and removal. As inflated spheroids deflated, GCaMP6s fluorescence gradually diminished to basal levels (Figure 4, C and D; Supplemental Video S4B). Collectively, these data highlight a potential role for calcium signaling in the regulation of macropinocytosis.

**Figure:** Video S4 A) Representative time-courses of calcium signaling in isolated body columns treated with DMSO (top-left), Jedi1 (200 μM; top-right), Jedi2 (200 μM; bottom-left), or ionomycin (10 μM; bottom-right), visualized by GCaMP6s. Corresponds to Figures 4A, 4B. Time stamps indicate time relative to drug addition; hh:mm:ss. B) Representative time-course of calcium signaling in spheroid during and after inflation and needle removal, visualized by GCaMP6s. “INFLATE” label marks period of inflation. Note the second calcium transient immediately after inflation period, corresponding to needle removal. Corresponds to Figures 4C, 4D. Time stamp indicates time relative to initiation of inflation; hh:mm:ss.

To further test the extent to which these effects were driven by calcium signaling, we treated threaded *Hydra* body columns with ionomycin to promote calcium influx in a stretch-activated channel-independent manner. Using GCaMP6s-expressing *Hydra*, we confirmed that ionomycin (calcium salt or free acid; 10 μM) induced an increase in fluorescence intensity and body column contractions, indicating an increase in cytosolic calcium concentrations (Figure 4, A and B; Supplemental Figure S4A; Supplemental Video S4A). In LifeAct-GFP-expressing body columns, ionomycin treatment (free acid; 10 μM) resulted in a decrease in macropinocytic cups, albeit more transient than Jedi1/2 treatment (Figure 4E). To determine whether calcium flux was necessary for ionomycin- and Jedi1/2-mediated inhibition of macropinocytosis, we repeated these experiments following calcium depletion. As calcium transients persisted in body columns following a number of singular treatments (unpublished data), we employed a combination of EGTA (2 mM), BAPTA-AM (30 μM), and Thapsigargin (1 μM) in deionized water to deplete extracellular and intracellular calcium stores (Nilsson *et al.*, 1998). Under these conditions, both ionomycin- and Jedi1/2-induced calcium transients were diminished (Supplemental Figure S4A). Importantly, calcium-depleted conditions abolished the ionomycin-mediated inhibition of macropinocytosis (Figure 4E). Jedi1/2, in contrast, retained the capacities to inhibit macropinocytosis in calcium-depleted samples (Figure 4E). Thus, while calcium influx is sufficient to transiently inhibit macropinocytosis, calcium flux alone cannot explain the effect observed upon Jedi1/2 treatment. Notably, comparable spheroid inflation experiments failed due to a prohibitively high incidence of spheroid rupture in calcium depleted conditions (unpublished data), precluding further analysis in this context.

## DISCUSSION

Here, we describe widespread macropinocytosis in the outer epithelium of *Hydra vulgaris*. Our results suggest that this phenomenon is regulated by tension applied to the epithelial layer. This is most directly demonstrated by the finding that inducing stretch in *Hydra* spheroids through inflation is sufficient to transiently inhibit macropinocytosis, with macropinocytosis rapidly recovering following deflation and relaxation of spheroids. This tissue stretch coincides with a rise in intracellular calcium in epithelial cells. We further show that pharmacological inhibition of stretch-activated channels results in the formation of macropinocytic cups and that the activation of stretch-activated channels and calcium ionophores repress macropinocytosis. Together, our findings highlight a role for tissue mechanics and stretch-activated channels in the regulation of macropinocytosis in *Hydra* (Figure 4F).

Macropinocytosis is historically categorized as “constitutive” or “stimulated,” based on the requirement for growth factors for ruffle induction. Constitutive macropinocytosis has been attributed to relatively few cell types, most notably, amoebae and antigen presenting cells, where respective roles in feeding and immune surveillance have been described (reviewed in Liu and Roche, 2015; King and Kay, 2019). In contrast, most mammalian epithelial models for which macropinocytosis has been reported require growth factor stimulation. Given that macropinocytosis in *Hydra* occurs in a wide variety of culture conditions without supplementation, our findings likely reflect an underappreciated form of constitutive epithelial macropinocytosis. Intriguingly, a similar ubiquitous macropinocytosis was recently reported in the epithelia of other cnidarian species (Ganot *et al.*, 2020), raising the possibility that constitutive macropinocytosis is more widespread than previously reported, at least among basal metazoans.

Our findings directly implicate cell surface tension as a regulator of macropinocytosis, expanding the repertoire of exogenous effectors beyond growth factors (West *et al.*, 1989) and pathogens (Rosales-Reyes *et al.*, 2012). Precisely how properties like cell surface tension may contribute to macropinocytosis remains unresolved. Membrane tension may be transduced to biochemical signals that limit actin nucleation and membrane protrusion. A similar mechanical-biochemical crosstalk has been described in the actin-mediated migration of neutrophils (Houk *et al.*, 2012; Diz-Muñoz *et al.*, 2016). Alternatively, macropinocytic cups may form and evolve more readily in low-tension conditions, as the energetic cost to deform membranes may be reduced (Aghamohammadazadeh and Ayscough, 2009). While we cannot exclude the latter possibility, our finding that the pharmacological inhibition of stretch-activated channels is sufficient to induce the formation of macropinocytic cups suggests an active contribution of stretch-activated channels in regulating this process.

In our experiments, gadolinium, which broadly inhibits stretch-activated channels, promoted the formation of macropinocytic cups. Moreover, application of the Piezo1 activators Jedi1/2 (Wang *et al.*, 2018) resulted in a transient inhibition of macropinocytosis, accompanied by calcium influx and tissue contraction. The efficacy of these drugs suggests a role for, but not limited to, stretch-activated channels in the regulation of the machinery underlying macropinocytic cup formation. Piezo function has been extensively explored in epithelia, where Piezo1 indirectly senses cellular crowding via membrane tension to maintain homeostatic cell densities (Eisenhoffer *et al.*, 2012; Gudipaty *et al.*, 2017). Intriguingly, recent work similarly identified Piezo1 as a regulator of epidermal growth factor-stimulated macropinocytosis in cancer cells (Kuriyama *et al.*, 2021). In mammalian myotubes, where a role for membrane tension in macropinocytosis has also been reported, tension-mediated redistribution of phospholipase D2 in the plasma membrane was proposed to promote actin remodeling and membrane ruffling, suggesting an alternative mechanism to transduce mechanical stimuli to biochemical signals (Lin and Liu, 2019; Loh *et al.*, 2019). Thus, tissue stretch may play a role in macropinocytosis in several contexts, and whether additional factors beyond stretch-activated channels contribute to this process in *Hydra* remains to be determined.

Stretch-activated channels provide a means to transduce mechanical stimuli to chemical signals by conducting ions across the plasma membrane (Coste *et al.*, 2010). While our findings using ionomycin suggest that calcium influx is sufficient to transiently inhibit macropinocytosis, differences in the effects of Jedi1/2, including more sustained inhibition in both control and calcium-depleted conditions, highlight the potential for additional regulation that cannot be accounted for by calcium alone. Piezos are nonselective cation channels capable of transporting various ions in addition to Ca^2+^, including K^+^, Na^+^, and Mg^2+^, that may contribute to their effects (Gnanasambandam *et al.*, 2015). While our efforts to deplete calcium also reduced the extracellular concentrations of other solutes, residual ions, including intracellular stores, may explain some effects of Jedi1/2 treatment. Alternatively, it is conceivable that the robust and sustained depolarization that occurs in the presence of ionomycin interferes with the mechanisms required to inhibit macropinocytosis. Lastly, we cannot ignore the possibility that Jedi1/2 may have off-target effects in *Hydra* that have yet to be elucidated. Future genetic studies will be necessary to explicitly implicate stretch-activated channels in macropinocytosis and its inhibition in *Hydra*.

Although both our pharmacological and physical perturbations implicate stretch in inhibiting macropinocytosis, we note several reasons for caution in interpreting these findings. For instance, calcium influx causes contraction of *Hydra*’s epithelial myonemes, which may, in turn, alter tension at the apical membrane. Thus, while Jedi1/2 treatment transiently inhibited macropinocytosis, these effects may be an indirect consequence of contraction. Moreover, calcium signaling plays a vital role in promoting exocytosis in diverse cell types (reviewed in Pang and Südhof, 2010). Notably, in lung epithelial cells grown on elastic substrates, the application of transient stretch is sufficient to promote calcium influx and enhance surfactant secretion (Wirtz and Dobbs, 1990). It is, therefore, conceivable that the inhibition of macropinocytosis we observe upon calcium influx may reflect enhanced secretion and a disruption of the counterbalancing forces of exo- and endocytic processes. Lastly, while resected body columns provide an unparalleled opportunity to observe macropinocytosis, their preparation requires substantially wounding tissues, which may contribute to the increase in macropinocytic cups we observe in this context. Nevertheless, our spheroid inflation approach circumvents acute injury on this scale and provides the most direct evidence for tissue mechanics in the regulation of macropinocytosis.

The role of macropinocytosis in *Hydra* remains unknown. Given *Hydra*’s predatory feeding behaviors, macropinocytosis is unlikely to play a significant role in nutrient acquisition. Similarly, the dilute solutes available in *Hydra*’s freshwater habitats likely render this process inefficient for acquiring or responding to dissolved environmental factors. Alternatively, macropinocytosis may contribute to the uptake and remodeling of contents at the animal’s surface. Notably, *Hydra* epithelia have been shown to internalize particles of various sizes, including beads of up to 1 μm in diameter (Technau and Holstein, 1992; Marchesano *et al.*, 2013), which demonstrates an ability to indiscriminately engulf solid substrates that may be explained by our findings. In one study, ultrastructural analysis revealed large, nanoparticle-filled vacuoles containing remains of the *Hydra* “cuticle,” a fibrous structure secreted by and surrounding *Hydra*’s apical ectodermal surface and home to a complex microbial community (Lentz, 1964; Böttger *et al.*, 2012; Marchesano *et al.*, 2013; Fraune *et al.*, 2015; He and Bosch, 2022). Thus, macropinocytosis may provide an efficient means to remodel the *Hydra* cuticle and sense and respond to the microbiome.

Given our data showing stretch-sensitivity, we also speculate that macropinocytosis may play a role in regulating membrane tension, as our findings suggest a significant capacity for apical membrane recycling. The *Hydra* epithelium is a remarkably dynamic tissue, characterized by perpetual cell proliferation, extensive cellular rearrangements ((Philipp *et al.*, 2009); reviewed in Campbell, 1974), and changes in tension during animal contraction and elongation. Based on measurements of macropinocytic cup size and macropinosome accumulation, we estimate rates of membrane retrieval in isolated body columns exceeding 150 μm^2^ per min per 10,000 μm^2^, corresponding to complete apical membrane turnover in ∼ 1 h. Despite this considerable membrane remodeling, we observed no apparent decrease in apical cell surface areas, suggesting comparable rates of membrane insertion in the epithelial cells of isolated body columns. Future efforts to uncouple membrane retrieval and insertion may shed light on a role for macropinocytosis in maintaining cell surface tension and, more broadly, reveal the contributions of tension-regulated macropinocytosis to *Hydra* physiology.

## MATERIALS AND METHODS

Request a protocol through *Bio-protocol*.

### *Hydra* culturing and strains

*Hydra* were maintained at 18°C in *Hydra* medium and fed 2–3 times per week with *Artemia* nauplii (Brine Shrimp Direct). Animals were starved ≥ 24 h before experimentation, and nonbudding animals were chosen for experimentation. The following transgenic lines were used:

DsRed2(ectoderm)/GFP(endoderm) (Glauber *et al.*, 2013)
LifeAct-GFP(ectoderm) (Aufschnaiter *et al.*, 2017)
pActin::GCaMP6s(ectoderm) (Szymanski and Yuste, 2019)
AEP SS1 (courtesy of Rob Steele)

### Microscopy

Experiments corresponding to Figures 2D and 4E were acquired on a Yokogawa CSU-W1 spinning disk confocal attached to an inverted Nikon Eclipse Ti2 microscope, with Hamamatsu C14440-20UP CMOS camera, using a 20X Plan Apo λ 0.75 NA objective. All other immunofluorescence and live images of LifeAct-expressing spheroids and body columns were acquired on a Yokogawa CSU100 spinning disk confocal attached to an inverted Nikon Ti-E microscope, with Hamamatsu C9100-13 EMCCD camera, using 20X Plan Apo VC 0.75 NA or 60XA Plan Apo VC 1.20 NA WI objectives. Images of GCaMP6s-expressing spheroids were acquired on an inverted Nikon Ti-E microscope with Lumencor SpectraX epifluorescence module and Andor Zyla camera, using a 10X Plan Apo 0.45 NA objective. Images of GCaMP6s-expressing body columns were acquired on an inverted Zeiss Axiovert 200M microscope with Point Grey Chamelion3 Monochrome camera, using a 5X EC Plan-Neofluar 0.16 NA objective, excepting images corresponding to Supplemental Figure S4A, which were acquired on an inverted Zeiss AXIO Observer.D1 microscope with AxioCam MRm camera, using a 5X EC Plan-Neofluar 0.16 NA objective. Confocal Z-stacks were acquired at 2–10 µm step sizes for a total depth of 30–100 µm, at 4–60 s time intervals. Where epifluorescence was used, images were acquired at a fixed focal plane at 5–30 s intervals. All images were acquired using Micro-Manager software (Edelstein *et al*., 2010).

### Immunofluorescence

Immunofluorescence was performed as previously described with slight modification (Aufschnaiter *et al*., 2017). In brief, AEP SS1 *Hydra* were relaxed for 1 min in 2% urethane (Sigma, Catalogue# 51-79-6)/*Hydra* medium, fixed for 1 h in 4% paraformaldehyde (PFA; Electron Microscopy Sciences, Catalogue# 15714)/*Hydra* medium, washed 3x in phosphate-buffered saline (PBS; Life Technologies, Catalogue# 20012-027), permeabilized for 15 min in 0.5% Triton X-100 (Sigma, Catalogue# 9036-19-5)/PBS, blocked for 1 h in 1% bovine serum albumin (BSA; Sigma, Catalogue# A7906)/0.1% Triton X-100/PBS (blocking solution), stained for 1 h in Alexa 488-phalloidin (Invitrogen, Catalogue# A12379) diluted to 1:200 in blocking solution, washed 3x in PBS, and mounted between a glass slide and coverslip with ProLong Gold mounting medium (Invitrogen, Catalogue# P36930).

### Tissue manipulations

Threaded body columns were prepared by amputating the head and foot (peduncle) from *Hydra* with a scalpel and inserting a 5–10 mm length of 8–lb fishing line (Trilene SensiThin) through the exposed lumen. Body columns were allowed to stabilize for 15 min before chemical perturbations, but were otherwise imaged immediately after preparation and transfer to imaging vessels, which consisted of either 35 mm glass bottom dishes (MatTek, P35G-1.5-14-C) or 96-well glass bottom plates (MatriPlate, MGB096-1-2-LG-L).

Spheroids were prepared by removing the head and foot from *Hydra* and cutting the remaining body column into three to four rings, which were subsequently cut longitudinally into two to three sections. Dissected tissues were allowed to heal unperturbed for 12–24 h before experimentation. Microinjection/inflation was achieved using a micropipette mounted to a Narishige motor-driven micromanipulator (MM-94) via Narishige microscope mounting adaptor, injection holder, and universal joint (NN-H-4, HI-9, UT-2, respectively). Pipettes were pulled from Sutter Instrument capillary tubes (#B150-110-10) on a Sutter Instrument micropipette puller (P-1000). Fluid ejection was controlled by a syringe attached to the pipette. Inflations were performed over a period of 2–3 min, after which the micropipette was immediately removed. Only spheroids that remained intact (did not rupture) were used to quantify macropinocytic cup abundance/dynamics. For mock inflations, a micropipette was inserted into the spheroid for 3 min, without injection, before removal.

### Dextran uptake

To visualize macropinosomes and dextran uptake in threaded body columns (Figure 1, E–G), body columns were transferred to a solution containing pHrodo Red Dextran, 10,000 MW (Thermo Fisher Scientific, Catalogue# P10361) diluted to 5 μg/ml in *Hydra* medium immediately before image acquisitions. To determine the effects of GdCl_3_ on macropinocytosis in intact *Hydra*, animals were incubated for 20 min in a comparable pHrodo dextran solution prepared in either control *Hydra* medium or *Hydra* medium with GdCl_3_ (50 μM), washed in *Hydra* medium to remove excess dextran, anesthetized in 2% urethane, and imaged immediately to minimize artifacts due to the cytotoxic effects of urethane.

### Chemical perturbations

GdCl_3_ (Sigma, Catalogue# 439770) stock solution was prepared in *Hydra* medium and diluted to a final concentration of 50 μM. For immunofluorescence experiments, animals were directly transferred to either the 50 μM GdCl_3_ solution or fresh *Hydra* medium (HM control) and incubated for 15 min before fixation. For threaded body column experiments, GdCl_3_ was diluted directly into imaging chamber *Hydra* medium to a final concentration of 50 μM. For drug perturbations, Jedi1 (Sigma, SML2533), Jedi2 (Sigma, SML2532), and ionomycin (free acid; Sigma, 407950) stock solutions were prepared in DMSO and diluted as 2x stocks (in *Hydra* medium or calcium-depleted medium) directly into imaging chamber medium to final concentrations of 200, 200, and 10 μM, respectively. DMSO controls corresponded to the highest DMSO concentration present in drug treatments. For calcium-depletion experiments, calcium-depleted medium was prepared by dissolving EGTA (2 mM; Sigma, Catalogue# 324626), BAPTA-AM (30 μM; Tocris Bioscience, Catalogue# 2787), and Thapsigargin (1 mM; Cayman Chemical, Catalogue# 10522) in deionized water. Threaded body columns were equilibrated in control *Hydra* medium or calcium-depleted medium for 30 min before drug addiction.

### Quantification and Statistical Analysis

Measurements of macropinocytic cup sizes were performed in FIJI using the built-in measure function for a line segment drawn along the long axis of macropinocytic cups at their maximum width. Measures of macropinocytic cups per cell were obtained from manual counts of macropinocytic cups and cells occupying the microscope field of view at a given time point. Quantifications assigned to the head, body column, and foot were generated from images obtained from the top, middle, and bottom one-third of the animal’s body length (tentacles excluded). Quantification of macropinosomes was similarly obtained by manual counts of dextran-filled puncta at a given time point. Average GCaMP6s fluorescence intensity was quantified by generating binary masks corresponding to body columns or spheroids in each frame, applying masks to unadulterated images to define regions of interest (ROIs), and quantifying the mean gray value of all pixels within the specified ROI using FIJI’s built-in measure function. The estimated fold change in spheroid volume before rupture was determined by approximating each spheroid as a true sphere and using the manually measured radius along the spheroid long axis before and after inflation (until rupture) for calculations.

## Supplementary Material





## Abbreviations used:

BAPTA-AM: 1,2-Bis(2-aminophenoxy)ethane- *N*, *N*, *N*’, *N*’-tetraacetic acid tetrakis (acetoxymethyl ester)
Ca ^2+^: calcium ion
EGTA: ethyleneglycol-bis(β-aminoethyl)- *N*, *N*, *N*’, *N*’-tetraacetic acid
GdCl3: gadolinium chloride
GFP: green fluorescent protein
GTPase: guanosine triphosphatase
HM: *Hydra* medium
PBS: phosphate-buffered saline
PFA: paraformaldehyde
ROI: region of interest
TRP: transient receptor potential

## References

[B1] Aghamohammadazadeh S Ayscough KR (2009). Under pressure: The differential requirements for actin during yeast and mammalian endocytosis. *Nat Cell Biol* *11*, 1039–1042. 19597484 10.1038/ncb1918 PMC2875176

[B2] Aufschnaiter R Wedlich-Söldner R Zhang X Hobmayer B (2017). Apical and basal epitheliomuscular F-actin dynamics during *Hydra* bud evagination. *Biol Open* *6*, 1137–1148. 28630355 10.1242/bio.022723 PMC5576072

[B3] Beams HW Kessel RG Shih C-Y (1973). The surface features of *Hydra* as revealed by scanning electron microscopy. *Trans Am Microsc Soc* *92*, 161.

[B4] Bloomfield G Kay RR (2016). Uses and abuses of macropinocytosis. *J Cell Sci* *129*, 2697–2705. 27352861 10.1242/jcs.176149

[B5] Bloomfield G Traynor D Sander SP Veltman DM Pachebat JA Kay RR (2015). Neurofibromin controls macropinocytosis and phagocytosis in Dictyostelium. *eLife* *4*, e04940. 25815683 10.7554/eLife.04940 PMC4374526

[B6] Böttger A Doxey AC Hess MW Pfaller K Salvenmoser W Deutzmann R Geissner A Pauly B Altstätter J Münder S, et al. (2012). Horizontal gene transfer contributed to the evolution of extracellular surface structures: The freshwater polyp *Hydra* is covered by a complex fibrous cuticle containing glycosaminoglycans and proteins of the PPOD and SWT (Sweet Tooth) families. *PLoS One* *7*, e52278. 23300632 10.1371/journal.pone.0052278 PMC3531485

[B7] Buccione R Orth JD McNiven MA (2004). Foot and mouth: Podosomes, invadopodia and circular dorsal ruffles. *Nat Rev Mol Cell Biol* *5*, 647–657. 15366708 10.1038/nrm1436

[B8] Buckley CM King JS (2017). Drinking problems: Mechanisms of macropinosome formation and maturation. *The FEBS Journal* *284*, 3778–3790. 28544479 10.1111/febs.14115

[B9] Campbell RD (1974). Cell movements in *Hydra*. *American Zoologist* *14*, 523–535.

[B10] Chiasson-MacKenzie C Morris ZS Liu C-H Bradford WB Koorman T McClatchey AI (2018). Merlin/ERM proteins regulate growth factor-induced macropinocytosis and receptor recycling by organizing the plasma membrane:cytoskeleton interface. *Genes Dev* *32*, 1201–1214. 30143526 10.1101/gad.317354.118 PMC6120716

[B11] Clayton EL Evans GJO Cousin MA (2008). Bulk synaptic vesicle endocytosis is rapidly triggered during strong stimulation. *J Neurosci* *28*, 6627–6632. 18579735 10.1523/JNEUROSCI.1445-08.2008 PMC2588494

[B12] Commisso C Davidson SM Soydaner-Azeloglu RG Parker SJ Kamphorst JJ Hackett S Grabocka E Nofal M Drebin JA Thompson CB, et al. (2013). Macropinocytosis of protein is an amino acid supply route in Ras-transformed cells. *Nature* *497*, 633–637. 23665962 10.1038/nature12138 PMC3810415

[B13] Coste B Mathur J Schmidt M Earley TJ Ranade S Petrus MJ Dubin AE Patapoutian A (2010). Piezo1 and Piezo2 are essential components of distinct mechanically activated cation channels. *Science* *330*, 55–60. 20813920 10.1126/science.1193270 PMC3062430

[B14] Diz-Muñoz A Thurley K Chintamen S Altschuler SJ Wu LF Fletcher DA Weiner OD (2016). Membrane tension acts through PLD2 and mTORC2 to limit actin network assembly during neutrophil migration. *PLOS Biology* *14*, e1002474. 27280401 10.1371/journal.pbio.1002474 PMC4900667

[B54] Edelstein A Amodaj N Hoover K Vale R Stuurman N (2010). Computer control of microscopes using μManager. *Curr Protoc Mol Bio* *92*, 14.20.1–14.20.17. 10.1002/0471142727.mb1420s92 PMC3065365 20890901

[B15] Eisenhoffer GT Loftus PD Yoshigi M Otsuna H Chien C-B Morcos PA Rosenblatt J (2012). Crowding induces live cell extrusion to maintain homeostatic cell numbers in epithelia. *Nature* *484*, 546–549. 22504183 10.1038/nature10999 PMC4593481

[B16] Fraune S Anton-Erxleben F Augustin R Franzenburg S Knop M Schröder K Willoweit-Ohl D Bosch TC (2015). Bacteria–bacteria interactions within the microbiota of the ancestral metazoan Hydra contribute to fungal resistance. *ISME J* *9*, 1543–1556. 25514534 10.1038/ismej.2014.239 PMC4478695

[B17] Ganot P Tambutté E Caminiti-Segonds N Toullec G Allemand D Tambutté S (2020). Ubiquitous macropinocytosis in anthozoans. *eLife* *9*, e50022. 32039759 10.7554/eLife.50022 PMC7032929

[B18] Glauber KM Dana CE Park SS Colby DA Noro Y Fujisawa T Chamberlin AR Steele RE (2013). A small molecule screen identifies a novel compound that induces a homeotic transformation in *Hydra*. *Development* *140*, 4788–4796. 24255098 10.1242/dev.094490 PMC3833435

[B19] Gnanasambandam R Bae C Gottlieb PA Sachs F (2015). Ionic selectivity and permeation properties of human PIEZO1 channels. *PLoS One* *10*, e0125503. 25955826 10.1371/journal.pone.0125503 PMC4425559

[B20] Gudipaty SA Lindblom J Loftus PD Redd MJ Edes K Davey CF Krishnegowda V Rosenblatt J (2017). Mechanical stretch triggers rapid epithelial cell division through Piezo1. *Nature* *543*, 118–121. 28199303 10.1038/nature21407 PMC5334365

[B21] He J Bosch TCG (2022). *Hydra*’s Lasting Partnership with Microbes: The Key for Escaping Senescence? *Microorganisms* *10*, 774. 35456824 10.3390/microorganisms10040774 PMC9028494

[B22] Holt M Cooke A Wu MM Lagnado L (2003). Bulk membrane retrieval in the synaptic terminal of retinal bipolar cells. *J Neurosci* *23*, 1329–1339. 12598621 10.1523/JNEUROSCI.23-04-01329.2003 PMC6742272

[B23] Houk AR Jilkine A Mejean CO Boltyanskiy R Dufresne ER Angenent SB Altschuler SJ Wu LF Weiner OD (2012). Membrane tension maintains cell polarity by confining signals to the leading edge during neutrophil migration. *Cell* *148*, 175–188. 22265410 10.1016/j.cell.2011.10.050 PMC3308728

[B24] Kerr MC Teasdale RD (2009). Defining macropinocytosis. *Traffic* *10*, 364–371. 19192253 10.1111/j.1600-0854.2009.00878.x

[B25] King JS Kay RR (2019). The origins and evolution of macropinocytosis. *Philos Trans R Soc Lond B Biol Sci* *374*, 20180158. 30967007 10.1098/rstb.2018.0158 PMC6304743

[B26] Kuriyama M Hirose H Masuda T Shudou M Arafiles JVV Imanishi M Maekawa M Hara Y Futaki S (2021). Piezo1 activation using Yoda1 inhibits macropinocytosis and proliferation of cancer cells. *bioRxiv*, 10.1101/2021.05.14.444123.

[B27] Lentz TL (1964). *The cell biology of Hydra.* Yale Medicine Thesis Digital Library, http://elischolar.library.yale.edu/ymtdl/2849.

[B28] Lin S-S Liu Y-W (2019). Mechanical stretch induces mTOR recruitment and activation at the phosphatidic acid-enriched macropinosome in muscle cell. *Front Cell Dev Biol* *7*, 78. 31139627 10.3389/fcell.2019.00078 PMC6518962

[B29] Lin XP Mintern JD Gleeson PA (2020). Macropinocytosis in different cell types: Similarities and differences. *Membranes* *10*, 177. 32756454 10.3390/membranes10080177 PMC7463864

[B30] Liu Z Roche PA (2015). Macropinocytosis in phagocytes: Regulation of MHC class-II-restricted antigen presentation in dendritic cells. *Front Physiol* *6*, 1. 25688210 10.3389/fphys.2015.00001 PMC4311620

[B31] Loh J Chuang M-C Lin S-S Joseph J Su Y-A Hsieh T-L Chang Y-C Liu AP Liu Y-W (2019). An acute decrease in plasma membrane tension induces macropinocytosis via PLD2 activation. *J Cell Sci* *132*, jcs232579. 31391241 10.1242/jcs.232579

[B32] Marchesano V Hernandez Y Salvenmoser W Ambrosone A Tino A Hobmayer B de la Fuente MJ Tortiglione C (2013). Imaging inward and outward trafficking of gold nanoparticles in whole animals. *ACS Nano* *7*, 2431–2442. 23448235 10.1021/nn305747e

[B33] Mellström K Höglund A-S Nistér M Heldin C-H Westermark B Lindberg U (1983). The effect of platelet-derived growth factor on morphology and motility of human glial cells. *J Muscle Res Cell Motil* *4*, 589–609. 6685736 10.1007/BF00712117

[B34] Nilsson H Videbæk LM Toma C Mulvany MJ (1998). Role of Intracellular Calcium for Noradrenaline-Induced Depolarization in Rat Mesenteric Small Arteries. *JVR* *35*, 36–44. 10.1159/000025563 9482694

[B35] Orth JD Krueger EW Weller SG McNiven MA (2006). A novel endocytic mechanism of epidermal growth factor receptor sequestration and internalization. *Cancer Res* *66*, 3603–3610. 16585185 10.1158/0008-5472.CAN-05-2916

[B36] Pang ZP Südhof TC (2010). Cell Biology of Ca2+-Triggered Exocytosis. *Curr Opin Cell Biol* *22*, 496–505. 20561775 10.1016/j.ceb.2010.05.001 PMC2963628

[B37] Philipp I Aufschnaiter R Ozbek S Pontasch S Jenewein M Watanabe H Rentzsch F Holstein TW Hobmayer B (2009). Wnt/β-Catenin and noncanonical Wnt signaling interact in tissue evagination in the simple eumetazoan *Hydra*. *Proc Natl Acad Sci* *106*, 4290–4295. 19237582 10.1073/pnas.0812847106 PMC2646623

[B38] Rosales-Reyes R Pérez-López A Sánchez-Gómez C Hernández-Mote RR Castro-Eguiluz D Ortiz-Navarrete V Alpuche-Aranda CM (2012). Salmonella infects B cells by macropinocytosis and formation of spacious phagosomes but does not induce pyroptosis in favor of its survival. *Microb Pathog* *52*, 367–374. 22475626 10.1016/j.micpath.2012.03.007

[B39] Sallusto F Cella M Danieli C Lanzavecchia A (1995). Dendritic cells use macropinocytosis and the mannose receptor to concentrate macromolecules in the major histocompatibility complex class II compartment: Downregulation by cytokines and bacterial products. *J Exp Med* *182*, 389–400. 7629501 10.1084/jem.182.2.389 PMC2192110

[B40] Sarkar K Kruhlak MJ Erlandsen SL Shaw S (2005). Selective inhibition by rottlerin of macropinocytosis in monocyte-derived dendritic cells. *Immunology* *116*, 513–524. 16313365 10.1111/j.1365-2567.2005.02253.x PMC1802442

[B41] Sato-Maeda M Tashiro H (1999). Development of oriented motion in regenerating Hydra cell aggregates. *Jzoo* *16*, 327–334.

[B42] Shimizu H Sawada Y Sugiyama T (1993). Minimum tissue size required for Hydra regeneration. *Dev Biol* *155*, 287–296. 8432387 10.1006/dbio.1993.1028

[B43] Siebert S Farrell JA Cazet JF Abeykoon Y Primack AS Schnitzler CE Juliano CE (2019). Stem cell differentiation trajectories in *Hydra* resolved at single-cell resolution. *Science* *365*, eaav9314. 31346039 10.1126/science.aav9314 PMC7104783

[B44] Skokan TD Vale RD McKinley KL (2020). Cell sorting in *Hydra vulgaris* arises from differing capacities for epithelialization between cell types. *Curr Biol* *30*, 3713–3723.e3. 32795440 10.1016/j.cub.2020.07.035 PMC7541579

[B45] Stewart TA Davis FM (2019). Formation and function of mammalian epithelia: Roles for mechanosensitive PIEZO1 ion channels. *Front Cell Dev Biol* *7*, 260. 31750303 10.3389/fcell.2019.00260 PMC6843007

[B46] Szymanski JR Yuste R (2019). Mapping the whole-body muscle activity of *Hydra vulgaris*. *Curr Biol* *29*, 1807–1817.e3. 31130460 10.1016/j.cub.2019.05.012

[B47] Technau U Holstein TW (1992). Cell sorting during the regeneration of *Hydra* from reaggregated cells. *Dev Biol* *151*, 117–127. 1577184 10.1016/0012-1606(92)90219-7

[B48] Wang Y Chi S Guo H Li G Wang L Zhao Q Rao Y Zu L He W Xiao B (2018). A lever-like transduction pathway for long-distance chemical- and mechano-gating of the mechanosensitive Piezo1 channel. *Nat Commun* *9*, 1300. 29610524 10.1038/s41467-018-03570-9 PMC5880808

[B49] West MA Bretscher MS Watts C (1989). Distinct endocytotic pathways in epidermal growth factor-stimulated human carcinoma A431 cells. *J Cell Biol* *109*, 2731–2739. 2556406 10.1083/jcb.109.6.2731 PMC2115909

[B50] Wirtz HR Dobbs LG (1990). Calcium mobilization and exocytosis after one mechanical stretch of lung epithelial cells. *Science* *250*, 1266–1269. 2173861 10.1126/science.2173861

[B51] Yang X Sachs F (1989). Block of stretch-activated ion channels in Xenopus oocytes by gadolinium and calcium ions. *Science* *243*, 1068–1071. 2466333 10.1126/science.2466333

