# Supplementary material for: Mechanical stretch regulates macropinocytosis in Hydra vulgaris
Source: Mol Biol Cell. 2024 Feb 2;35(3):br9. doi: 10.1091/mbc.E22-02-0065 (PMC10916863; doi:10.1091/mbc.E22-02-0065)
Supplement: Supplementary file 5 [file mbc-35-br9-s001.pdf]

Supplemental Materials

Molecular Biology of the Cell

Skokan *et al.*

Supplemental Figure Legends

Figure S1. A) Schematic (left), representative images (middle), and quantification (right) of macropinocytic cup abundance in the head, body column, and foot of fixed, intact *Hydra* stained with phalloidin (bars: mean ± sd; n ≥ 18 animals from 3 independent sample preparations per body region; one-way ANOVA with Tukey's multiple comparisons test). All frames depict maximum intensity projections of 10–35 μm z-stacks. B) Quantification of macropinocytic cup abundance in fixed intact *Hydra* (n = 15 from 3 independent preparations) and live, threaded body columns (n = 4 independent sample preparations), reproduced from data in Fig. 2A, 2B (bars: mean ± sd).

Figure S2. Representative images of live, intact *Hydra* expressing LifeAct-GFP in the ectoderm, treated with control *Hydra* medium (left) or $GdCl_3$ (right; 50 μM for 15 min).

Figure S3. Quantification of the approximated fold change in spheroid volume achievable prior to tissue rupture during spheroid inflation (bars: mean ± sd; n = 11 from 4 independent sample preparations).

Figure S4. A) Quantification and corresponding traces depicting mean fluorescence intensity over time in threaded body columns following specified treatment (line and shading: mean ± sd). Quantification depicts the fold change in fluorescence intensity (relative to pretreatment values) averaged over a 10-min period immediately following drug addition (n ≥ 3 independent sample preparations per condition; two-way ANOVA with Sidak's multiple comparisons test). Traces are normalized to pretreatment values. B) Representative stills of threaded body columns expressing LifeAct-GFP in the ectoderm before (Pre-treatment) and 10 min after (Post-treatment) specified treatments. Body columns were incubated in control *Hydra* medium or calcium-depleted conditions prior to treatment. Scale bars, 20 μm.

Supplemental Video Legends

Video S1. A) Representative time-course of macropinocytic cup formation, closure, and dissipation, visualized by LifeAct-GFP. Image registration was performed to compensate for translational movement in the body column. Corresponds to Figure 1D. B) Representative time-course of fluorescently labeled dextran (magenta) engulfment by macropinocytic cups, visualized by LifeAct-GFP (white). Image registration was performed to compensate for translational movement in the body column. Corresponds to Figure 1E. (A, B) All videos depict maximum intensity projections of 10–50 μm z-stacks. Time stamps, hh:mm:ss.

Video S2. A) Representative time-course of macropinocytic cup frequency in a control isolated body column treated with *Hydra* medium (left) or $GdCl_3$ (50 μM; right), visualized by LifeAct-GFP. Corresponds to Figure 2A. B) Representative time-courses of macropinocytic cup frequency in isolated body columns treated with DMSO (top-left), Jedi1 (200 μM; top-right), Jedi2 (200 μM; bottom-left), visualized by LifeAct-GFP. Corresponds to Figures 2E, 2F. (A-D) All videos depict maximum intensity projections of 35–100 μm z-stacks. Time stamps indicate time relative to drug addition; hh:mm:ss.

Video S3. A) Representative time-course of spheroid inflation. Corresponds to Figure 3A. B) Representative time-course of macropinocytic cup frequency in spheroids imaged before (pre-puncture; top) and after mock inflation (left) or inflation (right), visualized by LifeAct-GFP. Note “pre-puncture” frames depict still images (single time point). Corresponds to Figures 3B, 3C. Videos in (B) depict maximum intensity projections of 35–100 μm z-stacks. Time stamp indicates time relative to needle removal following treatment; hh:mm:ss.

Video S4. A) Representative time-courses of calcium signaling in isolated body columns treated with DMSO (top-left), Jedi1 (200 μM; top-right), Jedi2 (200 μM; bottom-left), or ionomycin (10 μM; bottom-right), visualized by GCaMP6s. Corresponds to Figures 4A, 4B. Time stamps indicate time relative to drug addition; hh:mm:ss. B) Representative time-course of calcium signaling in spheroid during and after inflation and needle removal, visualized by GCaMP6s. “INFLATE” label marks period of inflation. Note the second calcium transient immediately after

inflation period, corresponding to needle removal. Corresponds to Figures 4C, 4D. Time stamp indicates time relative to initiation of inflation; hh:mm:ss.

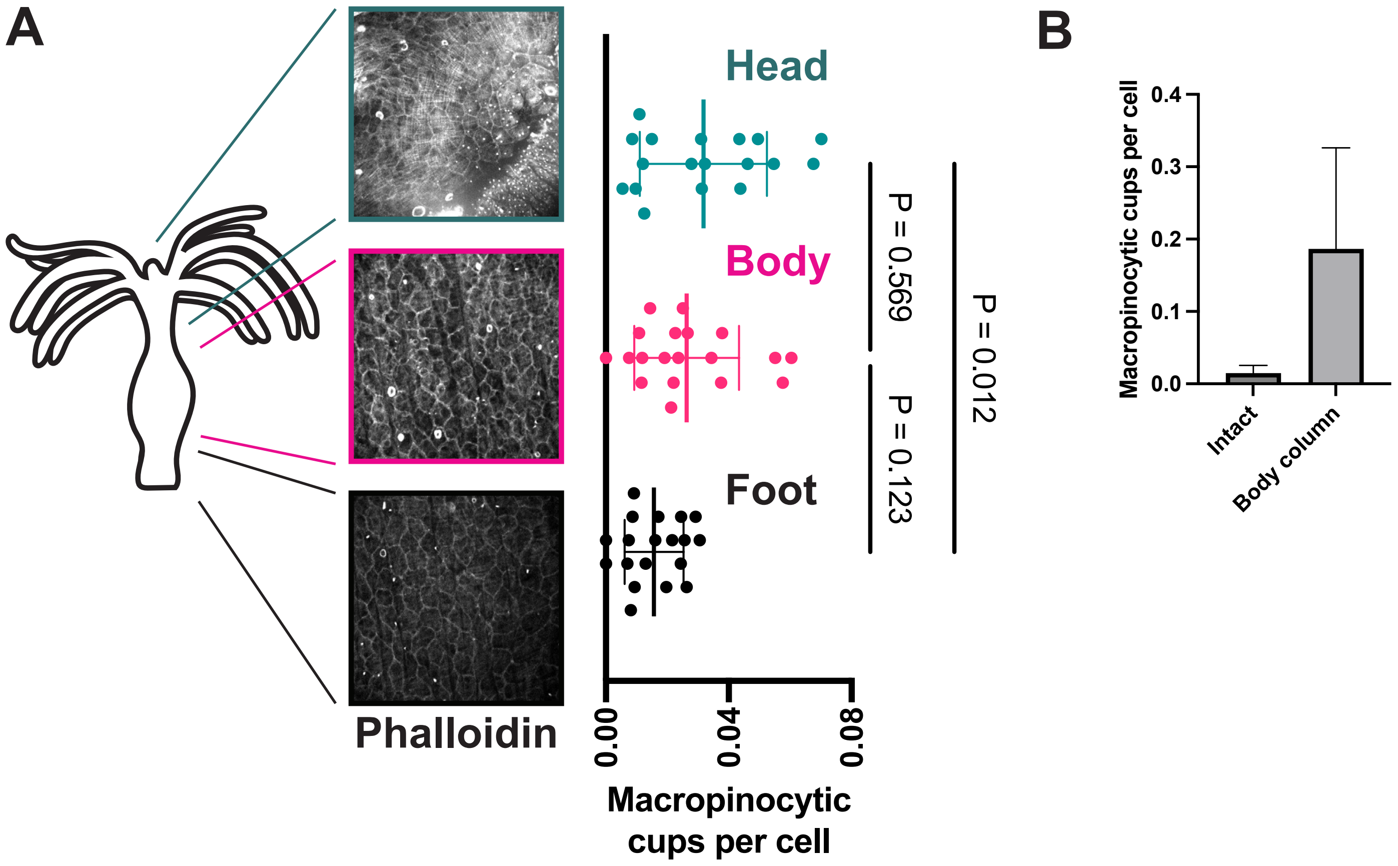
A
Head
Body
Foot
Phalloidin
P = 0.569
P = 0.123
P = 0.012
0.00
0.04
0.08
Macropinocytic cups per cell
B
0.4
0.3
0.2
0.1
0.0
Macropinocytic cups per cell
Intact
Body column

Figure S1.

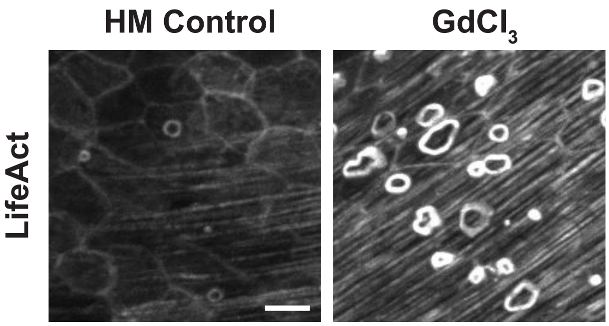

Figure S2.

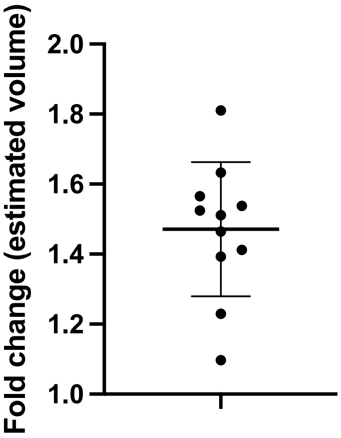

Figure S3.

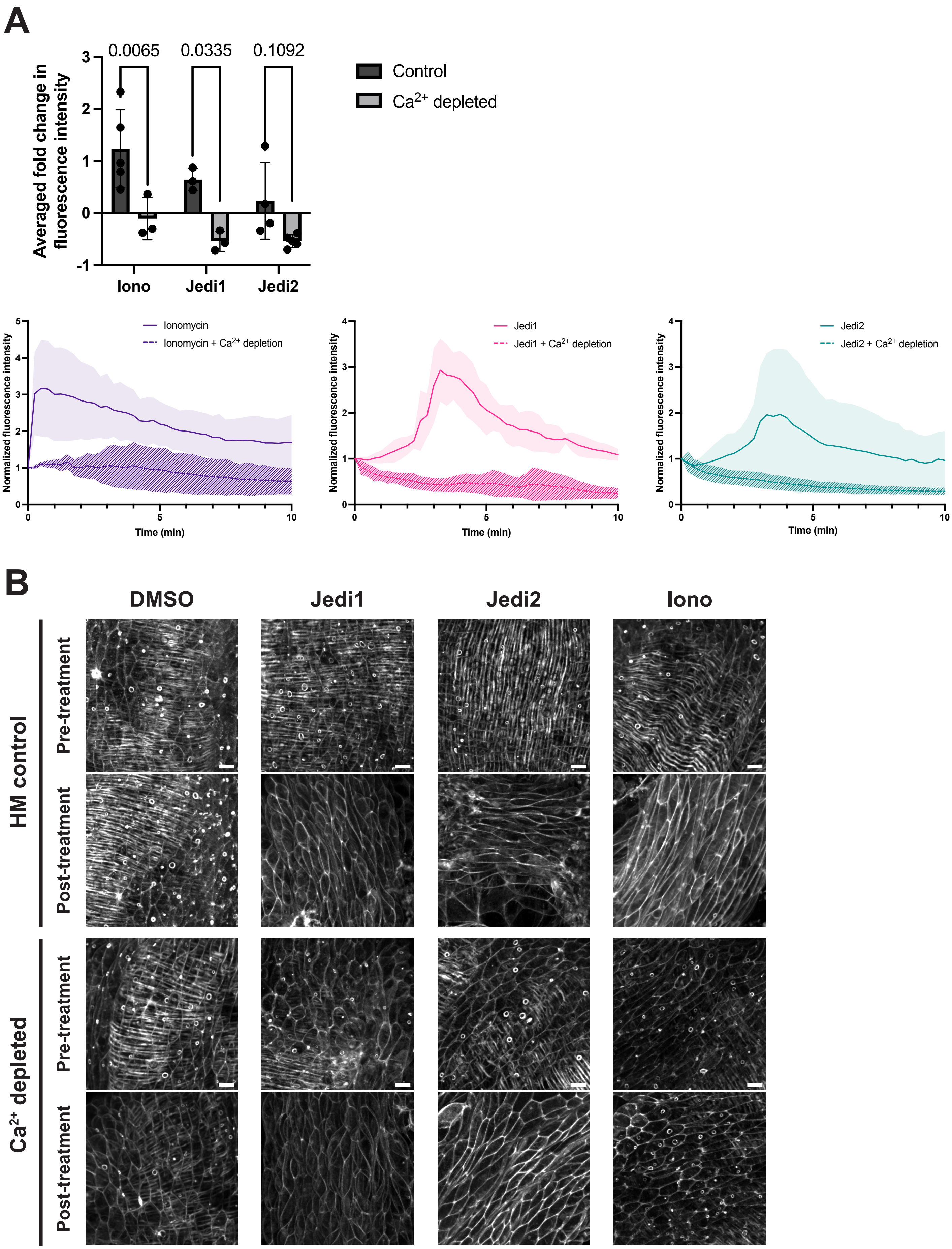

A
Averaged fold change in fluorescence intensity
0.0065
0.0335
0.1092
Control
Ca2+ depleted
Iono
Jedi1
Jedi2
Normalized fluorescence intensity
Time (min)
Ionomycin
Ionomycin + Ca2+ depletion
Jedi1
Jedi1 + Ca2+ depletion
Jedi2
Jedi2 + Ca2+ depletion
B
DMSO
Jedi1
Jedi2
Iono
HM control
Ca2+ depleted
Pre-treatment
Post-treatment

Figure S4.
