# Supplementary material for: Mechanical stretch regulates macropinocytosis in Hydra vulgaris
Source: Mol Biol Cell. 2024 Feb 2;35(3):br9. doi: 10.1091/mbc.E22-02-0065 (PMC10916863; doi:10.1091/mbc.E22-02-0065)
Supplement: Supplementary file 6 [file mbc-35-br9-s002.pdf]

Mechanical stretch regulates macropinocytosis in Hydra vulgaris

Taylor Skokan, Bert Hobmayer, Kara McKinley, and Ronald Vale

Corresponding author(s): Ronald Vale, University of California, San Francisco; Howard Hughes Medical Institute Janelia Research Campus

Review Timeline:

Submission Date:	2022-03-07
Editorial Decision:	2022-03-10
Revision Received:	2024-01-10
Accepted:	2024-01-17

Editor-in-Chief: Matthew Welch

Transaction Report:

(Note: With the exception of the correction of typographical or spelling errors that could be a source of ambiguity, letters and reports are not edited. The original formatting of letters and referee reports may not be reflected in this compilation.)

RE: Manuscript #E22-02-0065
TITLE: Mechanical stretch regulates macropinocytosis in Hydra vulgaris

Dear Dr. Vale:

Thanks for submitting this fascinating paper. I think that the plan for revision, stemming from the pre-print review at Review Commons seems like a sound one and should clear up any of the issues raised within the scope that you would like to address. One thing I would caution is that gadolinium is a very non-specific SAC inhibitor. Therefore, it would be best to refrain from using 'Piezo' unless that is the only SAC expressed in Hydra.

Sincerely,

Jody Rosenblatt
Monitoring Editor
Molecular Biology of the Cell

Dear Dr. Vale,

The review of your manuscript, referenced above, is now complete. The Monitoring Editor has decided that your manuscript is not acceptable for publication at this time, but may be deemed acceptable after specific revisions are made, as described in the Monitoring Editor's decision letter above and the reviewer comments below.

A reminder: Please do not contact the Monitoring Editor directly regarding your manuscript. If you have any questions regarding the review process or the decision, please contact the MBoC Editorial Office (mboc@ascb.org).

When submitting your revision include a rebuttal letter that details, point-by-point, how the Monitoring Editor's and reviewers' comments have been addressed. (The file type for this letter must be "rebuttal letter"; do not include your response to the Monitoring Editor and reviewers in a "cover letter.") Please bear in mind that your rebuttal letter will be published with your paper if it is accepted, unless you haveopted out of publishing the review history.

Authors are allowed 180 days to submit a revision. If this time period is inadequate, please contact us at mboc@ascb.org.

Revised manuscripts are assigned to the original Monitoring Editor whenever possible. However, special circumstances may preclude this. Also, revised manuscripts are often sent out for re-review, usually to the original reviewers when possible. The Monitoring Editor may solicit additional reviews if it is deemed necessary to render a completely informed decision.

In preparing your revised manuscript, please follow the instruction in the Information for Authors (www.molbiolcell.org/info-for-authors). In particular, to prepare for the possible acceptance of your revised manuscript, submit final, publication-quality figures with your revision as described.

To submit the rebuttal letter, revised manuscript, and figures, use this link: Link Not Available

Please contact us with any questions at mboc@ascb.org.

Thank you for submitting your manuscript to Molecular Biology of the Cell. We look forward to receiving your revised paper.

Sincerely,

Eric Baker
Journal Production Manager
MBoC Editorial Office
mbc@ascb.org

Full Revision

Review COMMONS

Manuscript number: RC-2021-01183
Corresponding author(s): Ron Vale, Kara McKinley

1. General Statements

Together with my co-authors, I wish to thank our reviewers and Managing Editor at Review Commons, Dr. Monaco, as well as our Monitoring Editor at MBoC, Dr. Rosenblatt, for their consideration of our manuscript, entitled **Mechanical stretch regulates macropinocytosis in *Hydra vulgaris***. After carefully considering the reviews solicited by Review Commons, we have addressed a number of points directly related to the conclusions put forth in our manuscript. In brief, our revisions primarily sought to 1) clarify the role of calcium in regulating macropinocytosis, 2) include a more direct metric for macropinocytosis, using a standard dextran-uptake assay, and 3) minimize our speculation regarding a functional role in regulating membrane tension. Our revisions are outlined in detail below, with a point-by-point response to the reviewer comments.

A number of reviewer suggestions are aimed at further probing the physiological role of macropinocytosis in *Hydra* or providing additional mechanistic dissection to elucidate other potential contributors to this phenomenon. While many of these recommendations warrant future investigation, we believe they would not change the findings of our current study and, therefore, fall outside the scope of this work. These too are addressed in detail below.

2. Point-by-point description of the revisions

Reviewer 1:

Major comments:
Most importantly, the role of Ca2+ entry via stretch-activated channels and how this would inhibit macropinocytosis remains unclear. In fact, the findings are somewhat counterintuitive since stretch applied to the monolayer would increase membrane tension while Ca2+ influx would support membrane delivery and exocytosis, thereby restoring tensional homeostasis.

We appreciate this important point regarding the effects of calcium influx on membrane delivery and exocytosis shown in other systems. We have included a section in the discussion addressing potential counterbalancing forces of exocytosis and endocytosis (p. 10-11).

In Fig 3, the authors demonstrate that applied stretch to the epithelium increases cytosolic Ca2+ and decreases membrane tension as expected. But whether the Ca2+ influx is required for the loss of macropinocytosis is not clear. This can be tested by either chelating Ca2+ transients in the cytosol or depleting the cells of Ca2+ by inhibiting ER-resident Ca2+ pumps and removing Ca2+ from the medium. In fact, if the authors think that extracellular Ca2+ is the only issue to

Review COMMONS

arresting macropinocytosis, substituting Ca2+ for another divalent cation (or removing all divalent cations from the medium, should the epithelium be amenable to it for short periods of time) could be employed.

We agree that this follow-up was important to demonstrate the necessity of calcium in inhibiting macropinocytosis. We have now performed the requested calcium depletion experiments in combination with our pharmacological perturbations (Fig. 4E). Our findings reveal that calcium is indeed necessary for the ionomycin-mediated effects; intriguingly, however, calcium depletion does not abolish the Jedi1/2-mediated effects. We have included a discussion of this discrepancy in the Discussion section (p. 9).

The connection between [Ca2+]cyto and macropinocytosis is established by Jedi and ionomycin. In the case of ionomycin, the large and sustained increase [Ca2+]cyto, well beyond what could be expected in physiological conditions, leads to the loss of plasma membrane PIP2, PIP3, and membrane associated F-actin. Jedi1/2 are certainly more targeted, but it is difficult to attribute their effects to Piezo in this system. More worryingly, the Ca2+ influx in response to Jedi2 and especially Jedi1 occurs maximally after 10 min of exposure. Yet, the authors show the complete loss of macropinocytic cups after 10 min (Fig 2E). It's difficult to reconcile that the Ca2+ is the issue.

Thank you for expressing this concern. We agree that the data suggest considerable complexity that we cannot currently account for. We have further emphasized in our results and discussion the discordance between ionomycin and Jedi1/2 experiments, and have highlighted the potential for additional and/or orthogonal layers of regulation (p. 9).

The authors do not quantify macropinocytosis beyond Figure 1. Instead, they use "macropinocytic cups" as their surrogate for bona fide, sealed macropinosomes. Macropinocytosis can occur at different scales and different rates, so the authors should instead use the 70 kDa dextran as the gold standard in Figure 2.

We appreciate this suggestion. We have now included dextran-uptake assays to support measures of macropinocytic cup frequency in animals treated with $GdCl_3$ (Fig. 2D). This inclusion has revealed that rates of macropinocytosis do not in fact increase in $GdCl_3$-treated animals, despite the increase in macropinocytic cup abundance. As a result, we have altered our language throughout the paper to refer to effects on macropinocytic cup frequency/formation, as opposed to rates of macropinocytosis.

And as part of gold standard approaches, the authors would appease the macropinocytosis field if they tested the requirement for PI3K and Na H+ exchangers in Figure 1.

Thank you for this suggestion. We agree that a comprehensive examination of relevant inhibitors would make a valuable contribution to dissecting the molecular mechanisms of macropinocytosis in Hydra. Pilot experiments performed using the NHE inhibitor EIPA and the PI3K inhibitor

LY294002 failed to elicit any apparent effect in our experimental paradigms. Nevertheless, we believe that these data are inconclusive and incomplete without further investigation and troubleshooting, including establishing efficacy of these reagents in *Hydra*, and have therefore excluded them from discussion in the manuscript.

The appearance of the GCAMP6s in Figure 2F before given Jedi2 is interesting. Aside from the Ca2+ signal that appears where the Hydra has been severed, the Ca2+ through the epithelium appears very heterogeneous. Does this Ca2+ signal oscillate in the cells and/or across the epithelium? Since the authors are able to image the cytoskeleton and Ca2+ in this system, it would be interesting to determine any correlations in their kinetics.

This is an interesting point. Unfortunately, we currently lack the necessary transgenic line to simultaneously observe and record calcium transients and the actin cytoskeleton in the ectoderm and have found calcium indicator dyes commonly exploited in other model systems insufficient reporters in Hydra. Nevertheless, in independent experiments using singly labeled animals, we do observe oscillatory waves of calcium across the ectoderm (visible to some extent in the supplemental videos), with no obvious actin fluctuations in LifeAct-expressing animals.

Reviewer 2:

Major comments

1. In Fig 2, the importance of SAC and Ca2+ for macropinocytosis are addressed. However, only one SAC inhibitor was used, whereas Ca2+ concentration in Ionomycin treated Hydra remained high even after 60 min when macropinocytic cup density had recovered (Fig 2E and G). As the authors mentioned in the DISCUSSION, other SAC transported cations may be involved in and thus need to be tested. Simply, the medium depleted of specific cation or water containing specific cation could be used to monitor the requirement of each cation on Jedi2 treated Hydra.

We appreciate this suggestion and agree that these insights are important to determine the requirement for additional ions in the observed effects. In an attempt to deplete calcium from our experimental media, we have now included experiments showing that SAC activators inhibit macropinocytosis in *Hydra* incubated in deionized water (Fig. 4E), precluding further dissection of the role of additional cations in the perceived effect.

2. In Fig 3, the authors demonstrate that increased membrane tension leads to higher Ca2+ concentration and less macropinocytic cups in Hydras. The SAC inhibitors and EDTA (or calcium free buffer) used in Fig2 should be applied in the inflated regenerative spheroids to confirm that membrane tension inhibits macropinocytosis via SAC and Ca2+.

This is a great point, and one that we were eager to address. Unfortunately, analogous inflation experiments in the presence of Gd^{3+} (SAC inhibitor) have not been possible, as spheroids in this condition are prone to rupture with minimal inflation, suggesting an effect on spheroid integrity

and precluding proper comparisons to spheroids inflated in the absence of SAC inhibition. We have noted this observation in the text (p. 7).

3. The authors observed an increase of macropinocytic cups in both amputated Hydra and regenerative spheroids than intact animal (0.186 and ~0.3 compared to 0.015 cups per cell, Fig S1, 2E, 3C). Would the inflation or inhibition of macropinocytosis perturb spheroid regeneration or polarization/sorting? Authors have discussed several potential biological functions of macropinocytosis in Hydra, including tension homeostasis and surface remodeling that are important during spheroid regeneration. It will be worthy to examine if mild membrane tension increase or SAC activation would delay the sorting process of regenerating Hydra tissues.

This is an interesting question. However, given that macropinocytosis is observed in intact animals, and any detrimental outcomes of Gd^{3+} or Jedi1/2 treatment on regeneration would be difficult to attribute to changes in macropinocytosis alone, we believe that these experiments fall outside the scope of this work.

Minor points:
1. Fig 2C is the quantification results of 2B but include three sets of data (labeled as 1, 2, and 3) without explanation.

Thank you for bringing this to our attention. We have modified the figure and legend for clarity (p. 12).

2. Would amputation of one tentacle lead to local or global Ca2+ reduction and macropinocytosis in a Hydra?

Despite our interest in this topic, we have unfortunately not been able to successfully explore the effects of more localized wounding on macropinocytosis due to technical difficulties associated with immobilizing and imaging otherwise-intact, live Hydra.

Reviewer 3:

Major points:
1. The function of macropinocytosis in the Hydra is not known. The author postulates that it could be linked to a regulation of membrane area during animal contractions. However, one may wonder if the membrane cell surface really changes during contractions. I wonder if another explanation is possible: most of the organisms leaving in fresh water require an efficient mechanism to remove excess water that comes in the cells through osmosis. The hydra regular contractile movement are part of this, and I am wondering the macropinocytosis could be linked to this mechanism. Would the author be able to apply osmotic shocks, in particular hypertonic shocks, and see how it changes the formation rate and the dynamics of macropinosomes? On

the reverse, in paralyzed animal, I am wondering if macropinosomes are still formed? Results from these experiments may give a clue about the function of macropinocytosis in the Hydra.

We agree that the physiological relevance of macropinocytosis in Hydra is open to many alternative interpretations and appreciate these suggestions. In the course of our experiments (in both ion-depleted experiments now included, and hypertonic treatments not discussed), we have found that any impact of osmotic stress on macropinocytosis is transient. However, given the difficulty of experimentally uncoupling osmotic stress and cell/membrane tension, and that macropinocytosis involves the uptake of water (not the elimination), we have elected to not further pursue this line of questioning. Moreover, we have found that macropinocytosis still occurs in animals treated with a number of the variably effective anesthetics previously reported in the Hydra literature (e.g. linalool, chloretone) and are not aware of any agents sufficient to completely paralyze animals.

2. Because of the role of Piezo and other mechano-sensitive calcium channels, the author conclude that the factor that limits macropinocytosis is membrane tension. However, unless I am mistaken, actin cytoskeleton has also been involved in mechano-sensing channels, it could be that cortical tension, rather than membrane tension is playing a regulatory role. A direct proof of membrane tension (by measuring it) changes would be required to conclude as the authors do. The role of membrane tension versus macropinocytosis could be directly assessed using membrane tension probes such as FliptR or flipper probes. Otherwise, a less clearly defined term, that combines both cortical tension and membrane tension, such as cell surface tension or cell tension would be preferable.

Thank you for this suggestion. We agree that our experiments cannot distinguish between membrane and cortical tensions, which may themselves be coupled. We have revised the language referring to our own experiments throughout the manuscript to refer to the more ambiguous “cell surface tension.”

3. Number of macropinocytic cups(actin rings) per cell is used as a readout for rate of macropinocytosis. Yet in addition to the number of cups parameters the diameter increases in certain conditions such as GdCi3. It would ideally be interesting to show the changes in diameter of cups and how this varies per in different conditions. For example, in videos of Jedi1 treated body columns the cups seem bigger in size. Supporting experiments of monitoring macropinosomes via dextran uptake assays needs to be performed for quantifications a rate of change in macropinocytosis is proposed. Alternatively, dextran beads of different molecular sizes with different fluorophores could also be used to assess the differences in rate and volume of uptake via macropinocytosis in various conditions of this study.

We appreciate this suggestion. We have now included dextran-uptake assays to support measures of macropinocytic cup frequency in animals treated with $GdCl_3$ (Fig. 2D) and adjusted the language of the text accordingly. Please see our comment above addressing a similar concern for further discussion.

4. If membrane tension is altered upon dissecting Hydra fragments, would it make sense to study potential changes in macropinocytosis within the regenerating body column? Such as differences in actin ring formation in cells close to wound edges versus equatorial regions of regenerating body columns and spheroids?

This is a very interesting question. However, we believe that interpretations of any such observations would be severely limited without a means to directly quantify local differences in tension across the amputated body column, and, while worthy of future efforts, developing the necessary approaches is beyond the scope of this work.

5. Reasoning for selection of Piezo as molecular target over other stretch activated channels has not been provided. Piezo activators have been used, on the contrary depletion of Piezo via RNAi could be performed in intact animals to assess increased macropinocytosis. Furthermore, rate of macropinocytosis could be assessed in body columns generated from Piezo depleted animals. This would further support the direct role of Piezo in the process.

We appreciate this suggestion and agree that genetic experiments will be necessary to definitively demonstrate a role for Piezo in this process. However, achieving the extensive and robust knock down of gene expression necessary to ensure that expression is diminished in a patch of cells in question, while possible, is quite challenging in Hydra, typically requiring considerable time and effort to generate transgenic animals expressing siRNAs throughout the ectoderm. Further, systems for inducible knockout have been problematic in Hydra (Brooun et al., 2020), and it remains to be determined whether knockdown animals would be viable. We recognize that other SACs may play a role in this process, and have limited our explicit implication of Piezos and further emphasized broader effects on SACs throughout the manuscript.

Minor points:

1. The authors report differences in macropinocytosis based on different parts of the animal (Fig.S1), upon treating intact animals with GdCl3 (Fig.2C) how does this vary? Do the differences still persist in spite of increased macropinocytosis?

Thank you for this question. Given the modest differences in cup frequency observed along the body axis—only the foot vs. head comparison is statistically significant (adjusted P-value = 0.012; Tukey's multiple comparisons test)—we did not perform additional quantification by body region; however, qualitatively, Gd^{3+} treatment resulted in an increase in ring abundance across the animal, such that any quantifiable differences have limited potential to provide additional information to inform physiological relevance.

2. Hydra are animals with an elongated body column. Dissecting body columns of different lengths could give rise to spheroids of different volume, these could then be inflated to establish a comparative volume study with different volumes and macropinocytosis.

We appreciate this suggestion and agree with the sentiment that more quantifiable and/or scalable approaches to inflation will be worth considering for future studies. Given the low-throughput nature of our current inflation techniques, however, adding additional conditions/variables or attempts to further bin data quickly becomes prohibitive.

3. For all graphical representation it would also be ideal to state the p-values for each significant comparison to better appreciate differences instead of stars.

We have now included the complete p values in the figures as requested.

4. For better understanding of figures, highlight in graph legends and figure panels which tissue sample has been used i.e intact animal, body column or spheroid.

Thank you for this suggestion. We have revised the figures and figure legends to denote where specific sample preparations were employed.

5. A graphical representation could be given for the comparison of macropinocytic cups between intact hydra versus body column samples with statistical analysis. To appreciate the claims made by the authors regarding the trend of more cups being observed in body columns versus intact animals, as only the mean values are stated in the text.

We appreciate this suggestion; we have now included a graphical comparison (Fig. S1B).

6. In Fig.1F, there exist streaks of dextran distinctly outlining apical membranes of cell sets in the hydra epithelia, what are these suggestive of?

The dextran accumulated between cells and/or clusters of cells reflects local variations in the distance between the tissue's apical surface and the coverslip, as tissues in these experiments are not forced into direct contact with the glass. Dextran between the cell and glass is displaced to regions where apical surfaces are farther from the glass; accumulation at junctions highlights a convex apical cell surface.

7. Fig.S1 No p-values

Thank you for this note. We have adjusted the figure and figure legend to include indications of statistical significance.

RE: Manuscript #E22-02-0065R
TITLE: "Mechanical stretch regulates macropinocytosis in Hydra vulgaris"

Dear Ron and Kara,

I've had a chance to go over the revisions of your paper on Hydra and found it an interesting paper, aptly addressing the water aspect of this organism. I appreciated the ingenious experimental approaches, despite molecular limitations. I feel that you have addressed the reviewer commons suggested well, if not experimentally, by pointing out the limitations in your discussion. Congrats and happy new year.

Sincerely,
Jody Rosenblatt
Monitoring Editor
Molecular Biology of the Cell

Dear Dr. Vale:

Congratulations on the acceptance of your manuscript! Thank you for choosing to publish your work in Molecular Biology of the Cell (MBoC).

Within 10 days, an unedited PDF of your manuscript will be published on MBoC in Press, an early release version of the journal. The date your manuscript appears on this site is the official publication date.

Your copyedited and typeset manuscript will be scheduled for publication in the next available issue of MBoC. Our production team will notify you when the page proofs of your paper are ready for your review.

MBoC offers the option to publish your paper with immediate open access. Open access can increase the discoverability and usability of your research. If you would like to publish your paper with immediate open access but did not select this option during initial submission, please contact the MBoC Editorial Office (mbc@ascb.org).

We invite you to submit images related to your accepted manuscript to be considered for the journal cover.
Cover images must contain color, TIF Format
Size image at 7.4" wide x 5.85" deep
RGB and CMYK color (please submit two separate files, one in each format)
300 dpi resolution

Please also send a 1-paragraph description of the image that a general audience could understand. Include with the caption the name and institution of the individual(s) the image should be attributed to. Please provide this description in a Word file. You can send the file via email or by your favorite file transfer service to mboc@ascb.org.

Authors of Articles and Brief Communications are also encouraged to create a short video abstract to accompany their article when it is published. These video abstracts, known as Science Sketches, are up to 2 minutes long and will be published on YouTube and then embedded in the article abstract. Science Sketch Editors on the MBoC Editorial Board will guide you as you prepare your video. Information about how to prepare and submit a video abstract is available at www.molbiolcell.org/science-sketches. Please contact mboc@ascb.org if you are interested in creating a Science Sketch.

We look forward to publishing your paper in MBoC.
Sign up for MBoC's new content alerts here https://tinyurl.com/3bcsyk3e

Sincerely,

Eric Baker
Journal Production Manager
MBoC Editorial Office
mbc@ascb.org
